# Restaging magnetic resonance imaging of the rectum after neoadjuvant therapy: a practical guide

**DOI:** 10.1590/0100-3984.2024.0004

**Published:** 2024-07-16

**Authors:** Natally Horvat, João Miranda, Fernanda Kinochita, Tiago Lins de Carvalho, Giovanni Brondani Torri, Thiago José Pinheiro Lopes, Cesar Higa Nomura

**Affiliations:** 1 Department of Radiology, Memorial Sloan Kettering Cancer Center, New York, NY, USA.; 2 Faculdade de Medicina da Universidade de São Paulo (FMUSP), São Paulo, SP, Brazil.; 3 Department of Radiology, Brigham and Women’s Hospital, Harvard Medical School, Boston, MA, USA.; 4 Department of Radiology and Diagnostic Imaging, Universidade Federal de Santa Maria (UFSM), Santa Maria, RS, Brazil.; 5 Department of Radiology, Hospital Sírio-Libanês, São Paulo, SP, Brazil.

**Keywords:** Rectal neoplasms, Neoadjuvant therapy, Magnetic resonance imaging, Treatment outcome, Neoplasias retais, Terapia neoadjuvante, Ressonância magnética, Resultado do tratamento

## Abstract

Colorectal cancer is the third most common cancer and the second leading cause of cancer-related death. Rectal cancer accounts for approximately one-third of new colorectal cancer cases, with adenocarcinoma as the predominant subtype. Despite an overall decline in colorectal cancer incidence and mortality, due to advancements in screening, early diagnosis, and treatment options, there is a concerning increase in incidence rates among young patients. Recent significant advances in managing locally advanced rectal cancer, such as the establishment of different surgical approaches, neoadjuvant treatment using different protocols for high-risk cases, and the adoption of organ-preservation strategies, have increased the importance of the role played by radiologists in locoregional assessment on magnetic resonance imaging at baseline, at restaging, and during active surveillance of patients with rectal cancer. In this article, we review the role of restaging rectal magnetic resonance imaging after neoadjuvant therapy, providing radiologists with a practical, step-by-step guide for assessing treatment response.

## Abstract (pt)

O câncer colorretal é o terceiro câncer mais comum e a segunda principal causa de morte relacionada ao câncer. O câncer retal representa aproximadamente um terço dos novos casos de câncer colorretal, sendo o adenocarcinoma o subtipo predominante. Apesar de uma diminuição geral na incidência e mortalidade, impulsionada por avanços na prevenção do câncer, diagnóstico precoce e opções de tratamento aprimoradas, há uma preocupante elevação nas taxas entre os pacientes jovens. Avanços recentes significativos no manejo do câncer retal localmente avançado, como abordagens cirúrgicas, o uso de diferentes protocolos de tratamento neoadjuvante para casos de alto risco e a adoção de estratégias de preservação de órgãos, aumentaram o papel dos radiologistas na avaliação locorregional por meio da ressonância magnética na avaliação inicial, reestadiamento e vigilância ativa de pacientes com câncer retal. Este manuscrito tem como objetivo revisar o papel da ressonância magnética retal no reestadiamento após terapia neoadjuvante, fornecendo aos radiologistas um guia prático para revisar exames nesse contexto.

## INTRODUCTION

Colorectal cancer is the third most commonly diagnosed cancer in men and women and the second leading cause of cancer-related death worldwide^(1)^. More than 150,000 new cases of colorectal cancer are expected to be diagnosed in the United States in 2024, positioning the disease as a major contributor to cancer-related death^(2)^. Because of advances in screening, early diagnosis, and treatment, colorectal cancer mortality rates have decreased—from 29.2 deaths per 100,000 population in 1970 to 12.6 deaths per 100,000 population in 2020^(3)^. Nevertheless, there is a concerning rise in the proportion of individuals diagnosed with colorectal cancer, particularly rectal cancer, among individuals under 50 years of age^(4,5)^.

Currently, neoadjuvant therapy (NAT)—typically in the form of chemoradiation therapy (CRT)—followed by total mesorectal excision (TME) is considered the standard of care for locally advanced rectal cancer^(6,7)^. Depending on the histological findings after TME, some patients also undergo adjuvant systemic therapy. In recent years, nonoperative management (NOM) for selected patients with a complete response after NAT is an option that has increasingly been employed^(8,9)^. The aim of NOM is to avoid surgery (i.e., TME) while ensuring oncological safety and improving overall quality of life for patients.

It is possible to administer NAT in a different sequence, known as total neoadjuvant therapy (TNT), in which all systemic chemotherapy and CRT are administered before surgery. This newer approach is gaining prominence due to better rates of complete pathologic response (cPR) and patient outcomes, as shown in one meta-analysis^(8,9)^, and higher rates of nonsurgical cure—defined as a sustained complete clinical response (cCR)—as shown in the Organ Preservation in Patients With Rectal Adenocarcinoma (OPRA) trial^(10)^. Another new approach is programmed cell death 1 blockade immunotherapy in patients with locally advanced rectal cancer and mismatch repair deficiency; in an initial study, all patients who received this immunotherapy, with or without CRT, achieved a cCR^(11)^. These newer approaches may further increase the number of patients eligible for NOM.

Restaging rectal magnetic resonance imaging (MRI) plays a critical role in defining the response to NAT and helps the multidisciplinary team define the best next step (i.e., involving or avoiding surgery). In the case of surgery, restaging rectal MRI also provides a roadmap to determine the best surgical approach for complete surgical resection of all tumor sites. The aim of this article is to review the role of restaging rectal MRI after NAT, providing radiologists with a practical step-by-step guide for assessing treatment response.

## OVERVIEW OF NAT

Conventional NAT for locally advanced rectal cancer involves the concurrent administration of radiation and radiosensitizing agents. Typically, patients receive radiation therapy to the pelvic area after administration of a radiosensitizer, typically fluorouracil or capecitabine^(3)^. Following the completion of neoadjuvant CRT, patients typically undergo TME to remove the remaining tumor. After TME, adjuvant systemic chemotherapy may also be recommended to target any remaining cancer cells and reduce the risk of recurrence. Conventional NAT is a longstanding, effective strategy for the comprehensive management of locally advanced rectal cancer. After neoadjuvant CRT followed by TME, 20% of patients achieve a cPR^(12)^.

Although conventional NAT continues to be widely used as the standard of care for locally advanced rectal cancer, TNT, which involves the integration of systemic chemotherapy with neoadjuvant CRT before any TME, has been increasingly adopted and is experiencing rapid growth. There are two main types of TNT, depending on whether systemic chemotherapy is added before or after neoadjuvant CRT: induction chemotherapy followed by CRT; and consolidation chemotherapy administered after CRT. The most common systemic chemotherapies are as follows: folinic acid, fluorouracil, and oxaliplatin; capecitabine and oxaliplatin; and folinic acid, fluoracil, irinotecan, and oxaliplatin. Patients undergoing TNT exhibit lower rates of distal recurrence, better 3-year disease-free survival, improved compliance with therapy, superior overall survival, and notably higher rates of cPR^(3)^.

## ORGAN-PRESERVATION STRATEGY

Organ preservation in the treatment of rectal cancer involves maintaining rectal integrity through the avoidance of radical surgery. In 2004, Habr-Gama et al.^(14)^ devised an organ-preservation strategy for selected rectal cancer patients who achieved a cCR after NAT in Brazil. Since then, various studies have been published further exploring and validating their approach^(8)^. In rectal cancer, NOM is often referred to as a “watch-and-wait” approach or as active surveillance. Instead of undergoing TME immediately after NAT, patients undergoing NOM are monitored carefully through regular imaging and clinical assessments. Another organ-preservation approach involves local excision, utilizing either endoscopic microsurgery or transanal minimally invasive surgery, for selected patients with small viable lesions and an excellent response after NAT. The primary objective of organ-preservation strategies is to offer a individualized, minimally invasive alternative in the comprehensive management of rectal cancer. Such strategies emphasize effective disease control while minimizing the adverse effects associated with extensive surgery. This is especially significant in the case of low-rectal tumors, where abdominoperineal resection and permanent colostomy are conventionally indicated to provide an R0 resection (i.e., one in which the surgical margin is microscopically-negative for residual tumor). An organ-preservation approach seeks to tailor treatment to individual patient needs, thus optimizing outcomes and mitigating the impact of major surgical intervention^(9,14,15)^.

## ASSESSMENT OF THE RESPONSE TO NAT

Typically, the response to NAT is assessed 8—12 weeks after the initiation of the therapy, although the timing can vary depending on the treatment plan and trial^(8)^. Response is assessed through a multidisciplinary analysis incorporating endoscopy, digital rectal examination (DRE), and rectal MRI. The goal of a multidisciplinary response assessment is to classify the response as follows: complete clinical response (cCR), near-complete clinical response (nCR), or incomplete clinical response (iCR). The Memorial Sloan Kettering regression schema^(15)^ used in the OPRA trial^(16)^ continues to be employed in the ongoing Janus Rectal Cancer Trial; the category definitions can be summarized as follows:

– **cCR**
  - DRE: normal, without palpable tumor
  - Endoscopy: flat, white scar with telangiectasia, no ulcer, and no nodularities
  - MRI: no evidence of viable disease at the tumor bed or any sign of disease in general and no suspicious lymph nodes
– **nCR**
  - DRE: smooth induration or minor mucosal changes
  - Endoscopy: small mucosal nodules or minor mucosal abnormalities, superficial ulceration, mild persistent erythema, or scarring with telangiectasia
  - MRI: evidence of a very small volume of viable disease at the tumor bed or partial regression of lymph nodes
– **iCR**
  - DRE: palpable tumor
  - Endoscopy: clear viable tumor
  - MRI: clearly visible viable disease at the tumor bed or definitely suspicious lymph nodes

## RESTAGING RECTAL MRI

### MRI protocol

An optimal protocol should provide the necessary MRI sequences for response assessment while ensuring minimal acquisition time to prioritize patient comfort and ensure clinical efficiency.

#### Preparation

The use of an antispasmodic (e.g., glucagon or hyoscine butylbromide) shortly before the examination is beneficial to reduce motion artifacts (often observed in upper rectal tumors) caused by peristalsis. In addition, the use of a micro-enema (5 mL) can reduce susceptibility artifacts produced by rectal air^(17,18)^. Although the use of a micro-enema is controversial, it can easily be self-administered and can be particularly useful in the restaging setting, given that diffusion-weighted imaging (DWI) can be an important tool used as a complement to T2-weighted imaging (T2WI).

#### Protocol

The patient should be instructed to empty their bowels and bladder first and then lie comfortably in the supine position on the scanner bed. An MRI scanner with a field strength of 1.5 T or 3.0 T should be used and should be equipped with a phased-array surface coil that is adjusted to cover the region just below the pubic bone. Comparison with baseline scans is crucial for ensuring proper acquisition planning; it is especially important to select the axial oblique plane, which should be perpendicular to the tumor bed.

The main sequences to be acquired during restaging rectal MRI are two-dimensional fast spin-echo (FSE) T2WI without fat suppression and DWI (Table 1). In the restaging examination, as in the baseline examination, T2WI is fundamental and every effort should be made to ensure optimal T2WI quality. To assess the tumor bed^(19)^, extramural vascular invasion (EMVI), or tumor deposits, DWI is a valuable complement to T2WI^(20)^. Of note, the use of an endorectal coil, endorectal filling, sequences including T2WI with fat suppression, T1WI, and contrast-enhanced T1WI have not demonstrated added value in the local restaging of rectal cancer after NAT^(21)^.

**Table 1:** Restaging rectal MRI protocol.

Imaging technique	Details
Axial T2WI, large FOV	Whole pelvis, from the aortic bifurcation to the anal verge
Sagittal T2WI	Include both pelvic sidewalls
Axial oblique slice of the tumor bed	Perpendicular to the tumor bed, slice thickness of 3 mm
Coronal oblique slice of the tumor bed	Slice thickness of 3 mm
Coronal oblique slice of the anal canal	For lower rectal tumors, slice thickness of 3 mm
DWI*	With a b value ≥ 800 s/mm^2^ and including ADC maps

FOV, field of view.

* Two DWI sequences can be obtained: one with a large FOV of the pelvis and a low b value (≈ 800 s/mm^2^); and one with a small FOV perpendicular to the tumor bed and a higher b value (≈ 1500 s/mm^2^).

### Stepwise approach to reviewing restaging rectal MRI

#### Step 1 – Comprehensive review of the clinical history

The first step in reviewing a restaging rectal MRI examination is to perform a comprehensive review of the clinical history of the patient, including DRE findings, endoscopy findings, the type of NAT administered, and the time from the completion of NAT to the restaging rectal MRI^(22)^. Opinions vary regarding the optimal time from the completion of NAT to the first post-treatment restaging rectal MRI. The response assessment is typically performed 8–12 weeks after NAT completion, although the interval can be longer depending on the treatment approach; the optimal interval tends to be shorter after completion of CRT than after completion of TNT^(8,23)^.

#### Step 2 – Evaluation of the baseline MRI

Evaluation of the baseline MRI is important for radiologists to understand the precise location of the tumor bed and to identify any mucinous components, so as to avoid pitfalls (e.g., post-treatment changes that can mimic a viable tumor) and to identify extrarectal disease (e.g., extramural vascular invasion, tumor deposits, and lymph node invasion). For patients whose baseline MRI was performed at a different institution, it is highly recommended that patients and referring physicians be educated to provide the initial baseline rectal MRI in order to improve the interpretation of the restaging rectal MRI^(24)^.

#### Step 3 – Assessment of the treatment response

Assessment of the treatment response provides valuable data that correlate with patient outcomes and guides the next steps regarding disease management.

#### Treatment response and T2WI

Nonmucinous tumors will demonstrate a spectrum ranging from very low to intermediate signal intensity—corresponding to fibrosis and viable tumor tissue, respectively^(24)^. Mucinous tumors (i.e., those with > 50% mucin at baseline), tumors with mucinous features (i.e., those with < 50% mucin at baseline), and tumors undergoing mucinous/colloid degeneration (i.e., those beginning to produce mucin after NAT) may demonstrate different degrees of mucin content, fibrosis, and viable tumor tissue^(25)^. The mucin component demonstrates very high signal intensity on T2WI and can be either cellular or acellular on histopathology. Currently, MRI cannot distinguish between cellular and acellular mucin^(26)^.

#### Treatment response and DWI

Serving as a complement to T2WI, DWI can detect viable tumor in nonmucinous tumors^(27)^. Hypercellular tissues, such as viable tumors, restrict the movement of water molecules because of their dense interstitial space, resulting in high signal intensity on DWI and low signal intensity on apparent diffusion coefficient (ADC) maps. In contrast, fibrotic tissues, with their looser, collagenous matrix, allow freer movement of water molecules, leading to lower signal intensity on DWI and higher signal intensity on ADC maps. In addition, the morphological characteristics of residual tumors on DWI often reflect the geometry of the original tumors, such that employing a pattern-based approach for identifying residual disease can improve the diagnostic performance in predicting a complete response.

Interpreting DWI requires expertise, as evidenced by the moderate inter-reader agreement reported in the literature for determining the response to neoadjuvant CRT^(28)^, which improves slightly with the addition of T2WI (kappa = 0.402 vs. 0.51–0.688). Notably, the majority of positive DWI findings at restaging MRI align well with the endoscopic results, demonstrating a positive predictive value of 86%.

Viable non-mucinous tumor may appear as a focal wall thickening or nodules within the tumor bed, with high signal intensity on DWI and low signal intensity on ADC mapping. This differs from certain potential pitfalls, as outlined below.

**Figure 1:**
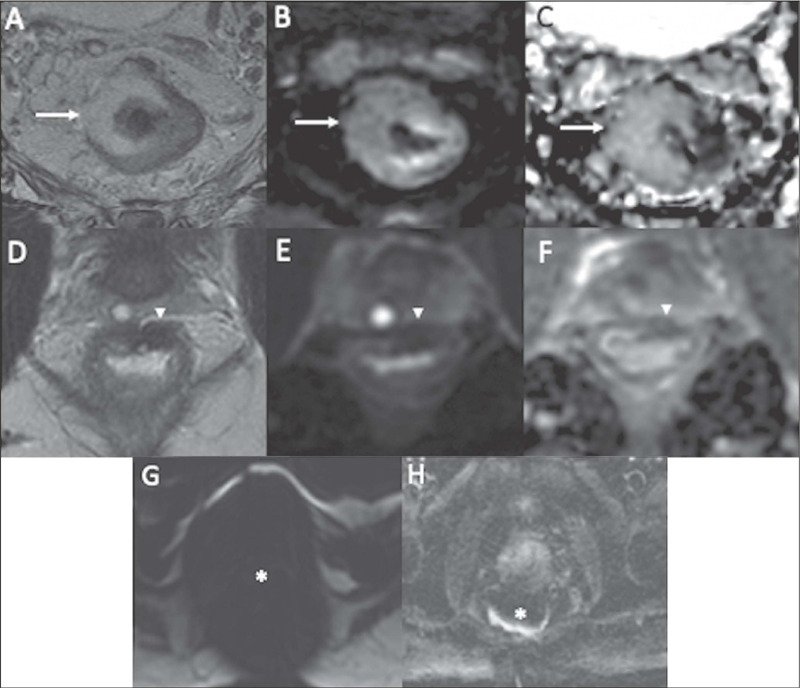
Examples of DWI artifacts. **A-C:** A T2 shine-through artifact (arrows) in a patient with a mucinous tumor showing high signal intensity on T2WI **(A),** DWI **(B),** and the ADC map **(C). D-F:** A T2 blackout artifact (arrowheads) identified by significant low signal intensity on T2WI **(D),** low signal intensity on DWI **(E),** and low signal intensity on the ADC map **(F),** representing fibrosis. **G,H:** Two additional examples of DWI artifacts (asterisks) due to surgical clips **(G)** and rectal air **(H).**

*Artifacts* – False positives (high signal intensity on DWI and low signal intensity on an ADC map) may result from susceptibility artifacts caused by rectal air or other artifacts like metal artifacts (Figure 1). As previously mentioned, rectal air artifacts can be minimized by administering a micro-enema before the examination^(17)^. If artifacts significantly compromise image quality, it is crucial to acknowledge this in the radiology report, and DWI should not be the basis for final interpretation.

*Lack of correspondence to the baseline tumor bed* – For DWI to be considered positive, it is essential that the suspicious area corresponds to the designated tumor bed. If the suspicious area is outside the designated tumor bed, it should not be regarded as indicating suspicion of a viable tumor.

*T2 shine-through* – The T2 shine-through effect occurs when there is high signal intensity on DWI and the ADC map, often representing fluid or mucin components. Intraluminal fluid typically shows T2 shine-through with a tri-radiate morphology (“Mercedes-Benz” sign), as depicted in Figure 1.

*T2 blackout* – The T2 blackout effect is identified by low signal intensity on DWI and the ADC map, representing fibrosis, and also shows markedly low signal intensity on T2WI^(24)^, as also depicted in Figure 1.

#### Treatment response classification

##### Post-NAT MRI tumor regression grade

The post-NAT magnetic resonance tumor regression grade (mrTRG) system is employed at some institutions. The mrTRG system is an adaptation of the TRG system that is used in pathology^(29)^. The post-NAT mrTRG generates a score from 1 to 5, based on the degree of tumor remaining and the amount of fibrosis after NAT, as detailed in Table 2. The total mrTRG score has been associated with disease-free and overall survival^(30)^, as well as having shown moderate accuracy for detecting a cPR^(31)^. Specifically, a post-NAT mrTRG score of 1 or 2 (indicating complete or substantial radiological regression, respectively) has been shown to have a sensitivity of 70–71% and a specificity of 62–68% for a cPR^(32,33)^. Although the use of the mrTRG system has shown some benefits, it is crucial to acknowledge the limited correlation between the mrTRG and pathologic TRG scores. In addition, the consistency in reading mrTRG scores varies significantly among different reviewers, with kappa values ranging from 0.25 to 0.80^(34,35,36)^. Furthermore, it is important to note that even when incorporating DWI into the mrTRG classification, the area under the receiver operating characteristic curve increased from 0.69 to only 0.74^(37)^.

**Table 2:** Post-NÄΓ mrTRG scoring.

Score	Description
mrTRGl	Minimal or no visible fibrosis (appearing as a thin linear scar), with low signal intensity on T2WI, and absence of tumor signal (intermediate signal intensity)
mrTRG2	Prominent fibrosis without tumor signal
mrTRG3	Mainly fibrotic but with noticeable, measurable areas of tumor signal
mrTRG4	Mostly tumor signal with negligible fibrosis
mrTRG5	Exclusive tumor presence or an increase in tumor size over baseline

##### cCR, nCR, and iCR

**Figure 2:**
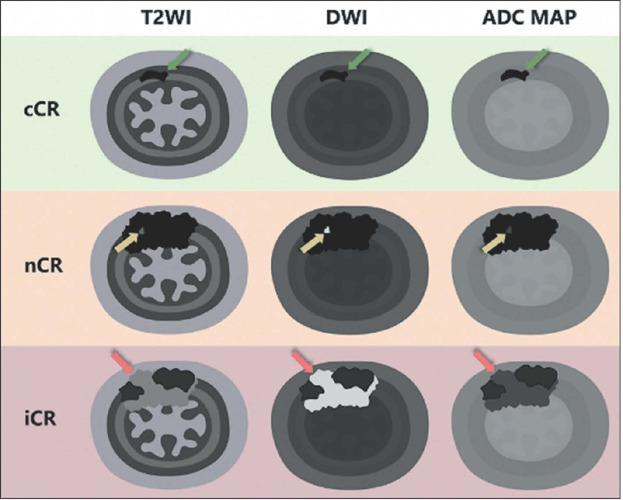
Illustration with examples of different clinical responses on restaging rectal MRI based on T2WI, DWI, and ADC mapping of the tumor bed. A cCR is characterized by markedly low signal intensity (SI) on T2WI and no restricted diffusion (low SI on DWI and the ADC map), indicated by green arrows. An nCR corresponds to marked fibrosis (low SI on T2WI, DWI, and the ADC map) with small areas of viable tumor, defined as intermediate SI on T2WI and restricted diffusion (high SI on DWI and low SI on the ADC map), indicated by yellow arrows. An iCR is defined as definite areas of viable tumor, indicated by red arrows.

The classification system used in the OPRA trial and recommended by some societies, such as the Society of Abdominal Radiology^(21)^, classifies treatment response into three groups (Figure 2): cCR, nCR, and iCR.

**Figure 3:**
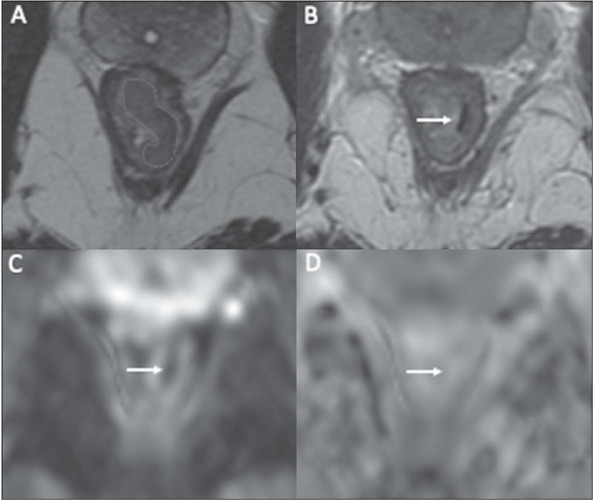
A cCR after NAT in a 41-year-old man with low-rectal adenocarcinoma. **A:** Baseline axial T2WI showing a low-rectal tumor (dotted line). **B:** Axial T2WI after the completion of NAT shows a thin hypointense scar at the site of the treated tumor (arrow). No diffusion restriction was present on DWI **(C)** or ADC mapping **(D).**

A cCR represents an extremely positive treatment response, defined as a significant reduction in tumor size, evidenced by marked disappearance of the intermediate signal on T2 restaging rectal MRI. Specific changes on T2WI and DWI, as depicted in Figure 3, include the following:

T2WI – There can be a linear or crescent-shaped scar in the mucosal/submucosal layers, or even a return of the rectal wall to a normal appearance. It is noteworthy that rectal wall normalization, which indicates a complete response, occurs in about 5% of cases^(38)^.

DWI – Absence of high signal intensity on images with a high b value^(19,39,40,41)^ Comparison with baseline images and referencing the normal rectum are crucial in this assessment. DWI is particularly useful for detecting cCR in small, subcircumferential scars^(27)^.

**Figure 4:**
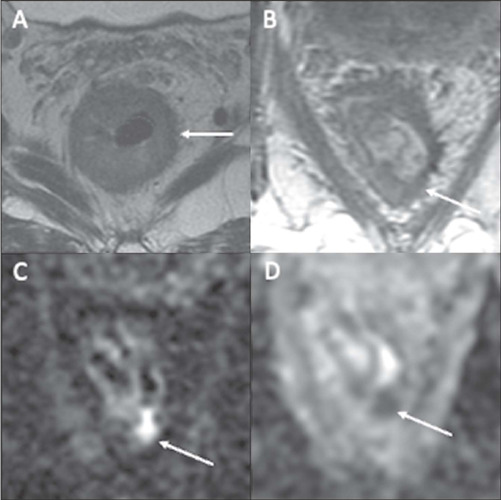
An nCR in a 63-year-old man with mid-rectal adenocarcinoma. **A:** Baseline axial T2WI showing a near circumferential low-rectal tumor with intermediate signal intensity (dotted line). **B:** Axial T2WI after the completion of neoadjuvant CRT showing new fibrosis and a residual posterior area with intermediate signal intensity on T2WI (arrow). **C:** Axial DWI showing high signal intensity (arrow) and axial ADC map **(D)** showing corresponding low signal intensity (arrow), suspicious for a small amount of viable tumor within the fibrotic tumor bed.

**Figure 5:**
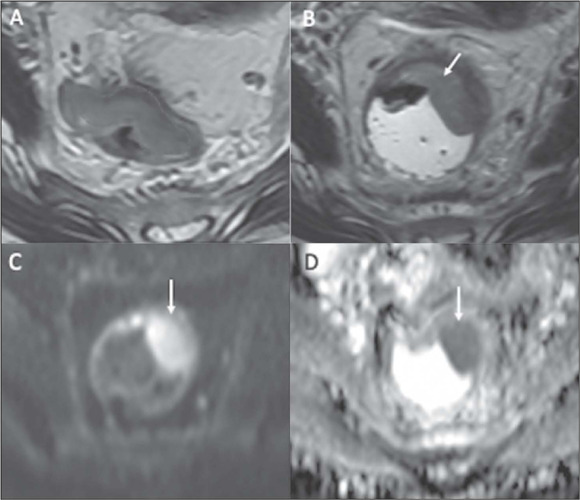
An iCR in a 39-year-old woman with mid-/high-rectal adenocarcinoma. **A:** Baseline axial T2WI showing intermediate signal intensity in a mid-/high-rectal tumor (dotted line). **B:** Axial T2WI after the completion of neoadjuvant CRT, showing persistent intermediate signal intensity (arrow), representing an iCR and residual tumor. Axial DWI **(C)** and ADC mapping **(D)** demonstrating restricted diffusion at the tumor site (arrows).

An nCR represents significant but not total regression. This category emerged from observations that many patients show a very good but not complete response and might achieve a cCR given more time between the completion of the NAT and the response assessment (Figure 4). With an nCR, there is a small area of intermediate signal intensity on T2WI or a small punctate area of restricted diffusion on DWI. An iCR represents significant residual tumor (Figure 5).

**Figure 6:**
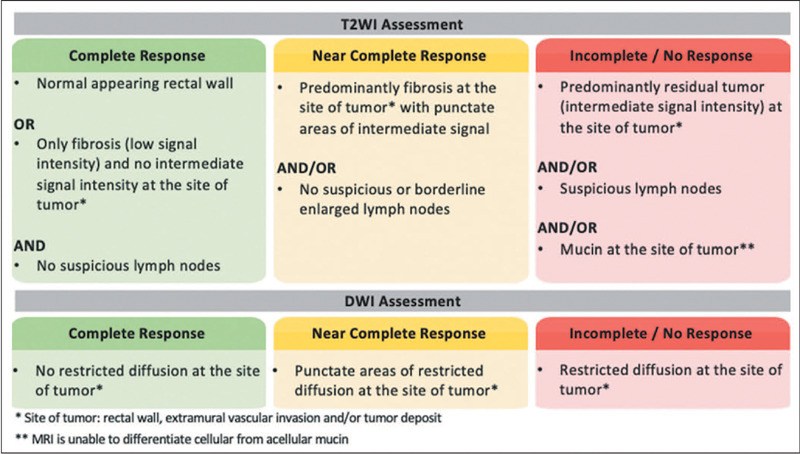
Summary of the final assessment on restaging rectal MRI.

If the institution and multidisciplinary team are aiming at organ preservation, defining the initial post-NAT response (cCR, nCR, or iCR) is important (Figure 6). Most patients with an initial cCR or nCR will have a sustained cCR; these patients are candidates for watch-and-wait management, potentially avoiding surgery^(42)^. However, patients with an iCR are not suited for watch-and-wait management^(15,16)^.

#### Step 4 – Evaluation of the relationship between the tumor and adjacent structures

In order to decide which surgical treatment is most appropriate for each patient, surgeons need to know whether or not the tumor has invaded the adjacent structures. It is also important to describe potential fibrotic changes and whether they involve adjacent structures. The following structures should be included when assessing treatment response: the mesorectal fascia (MRF), peritoneum, pelvic viscera (bladder, ureters, urethra, prostate, seminal vesicles, uterus, and vagina), pelvic sidewalls, iliac vessels, sciatic nerve, sacral roots, lumbosacral trunk, levator ani muscles, puborectalis muscle, external sphincter, intersphincteric space, internal sphincter, and pelvic bones.

The status of the MRF in particular is a crucial element. A clear MRF on restaging MRI has a positive predictive value of up to 90% for clear margins upon pathological examination^(43)^. Involvement of the MRF is evaluated by measuring the distance from the MRF to the nearest edge of the rectal tumor, taking into account the direct extent of the tumor, EMVI, tumor deposits, or lymph nodes with completely disrupted capsules^(44)^. Lymph nodes with intact capsules are not factored into the evaluation, because they do not typically increase the risk of local tumor recurrence. A distance of less than 0.1 cm is considered indicative of MRF involvement^(21)^. Although high-resolution T2WI plays a vital role in evaluating the involvement of the MRF, distinguishing between pure fibrosis and fibrotic tissue containing residual tumor cells can be challenging after NAT^(45)^.

#### Step 5 – Evaluation of the lymph nodes

In most cases, restaging rectal MRI will depict a notable reduction in lymph node size or complete resolution of the lymph node enlargement. Notably, the effectiveness of rectal MRI for nodal staging is significantly higher at restaging than at baseline. Restaging rectal MRI can identify patients with no residual nodal disease, with negative predictive values as high as 95%^(46)^. Unlike at baseline, when morphology is the most reliable parameter for evaluating the lymph nodes, lymph node morphology is an unreliable parameter at restaging. In contrast, the imaging finding that best correlates with pathology at restaging is the short-axis diameter of the lymph node^(21)^. Size criteria to identify suspicious lymph nodes are detailed in Table 3. At restaging, it is particularly important to evaluate the lateral pelvic lymph nodes, given that they are not routinely resected.

**Table 3:** Criteria for suspicious lymph nodes on restaging rectal MRI.

Location or aspect	Diameter (short axis)
Mesorectal, superior rectal	> 5 mm
Internal iliac	> 4 mm
Obturator	> 6 mm
Ml (inguinal, external iliac, common iliac, or retroperitoneal)	> 10 mm
Mucin within lymph nodes*	–

* MRI is unable to differentiate cellular from acellular mucin.

Adapted from Lee et al.^(21)^ under a Creative Commons Attribution 4.0 International License.

#### Step 6 – Evaluation of EMVI and tumor deposits

Given their association with poor prognosis, EMVI and tumor deposits should be thoroughly evaluated. The resolution of EMVI after NAT correlates with improved survival. Although it can be challenging to distinguish viable from nonviable tumor within EMVI, DWI has high specificity and a high negative predictive value for predicting a complete response within EMVI or a tumor deposit^(20)^. In cases of uncertainty, particularly if watch-and-wait management is being considered, multidisciplinary discussion is suggested and close follow-up might be indicated.

#### Step 7 – Providing a clinically meaningful conclusion

Lastly, providing a clinically meaningful conclusion is essential to guiding the multidisciplinary team in determining the optimal management plan after NAT. Figure 5 outlines the three common outcomes of restaging rectal MRI, considering T2WI and DWI. If the T2WI and DWI findings are discordant, it is recommended that the worse classification be considered. However, in such cases, the quality of the DWI should be taken into account.

## CONCLUSION

Restaging rectal MRI plays an important role in assessing the treatment response after NAT, helping the multidisciplinary team define the optimal post-NAT treatment plan that is tailored to the needs of the patient and will achieve the best outcome. A review of the clinical history and baseline rectal MRI of the patient, together with the use of a rectal MRI protocol that prioritizes relevant sequences and ensures correct axial oblique planes, are essential to providing a high-quality restaging rectal MRI. The T2WI sequence remains fundamental for categorizing the treatment response and for local staging; DWI serves as a complementary tool to enhance the certainty of interpretation. Clear communication of the treatment response classification and detailed descriptions of the structures involved are crucial for guiding the multidisciplinary team in choosing the best course of action, whether it involves a watch-and-wait approach or surgical resection.
